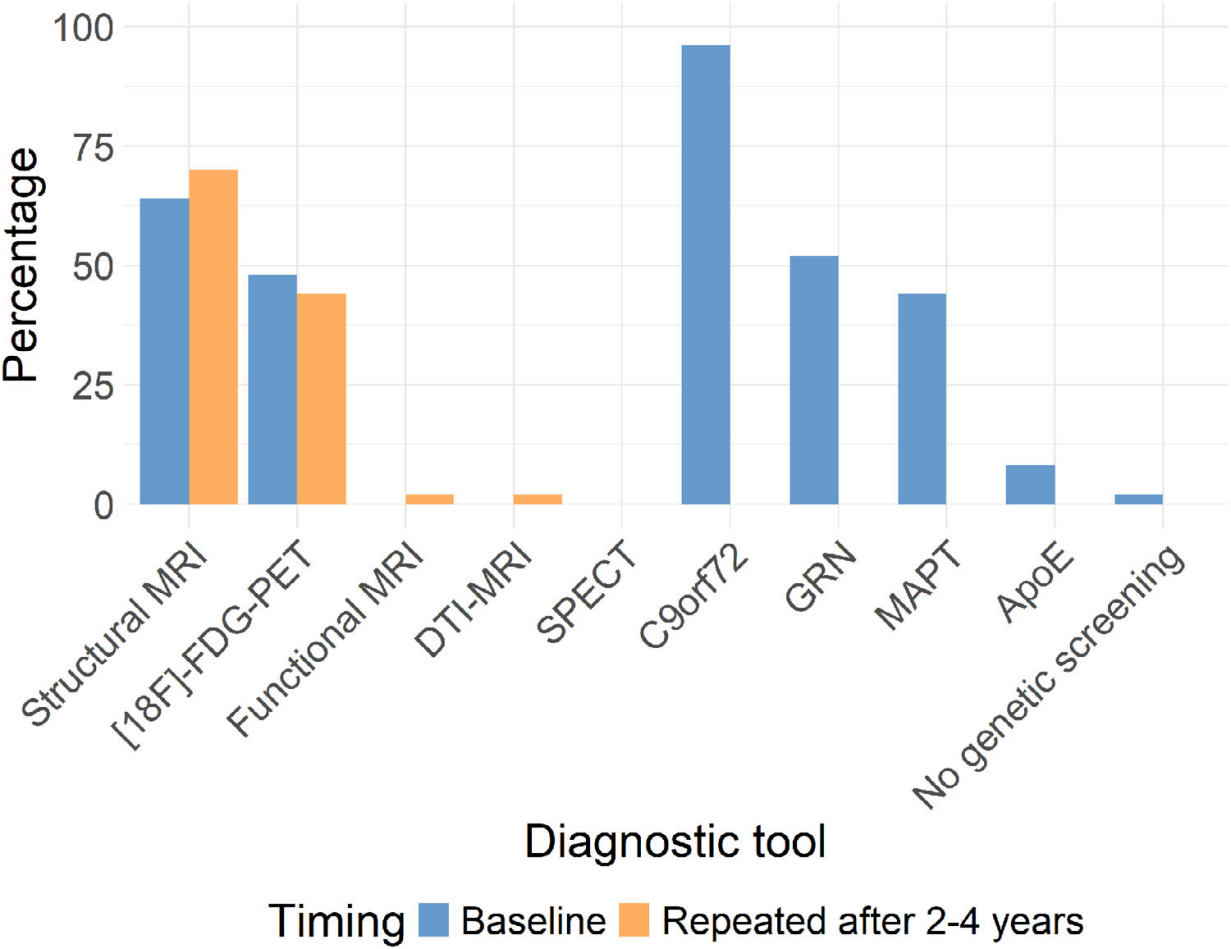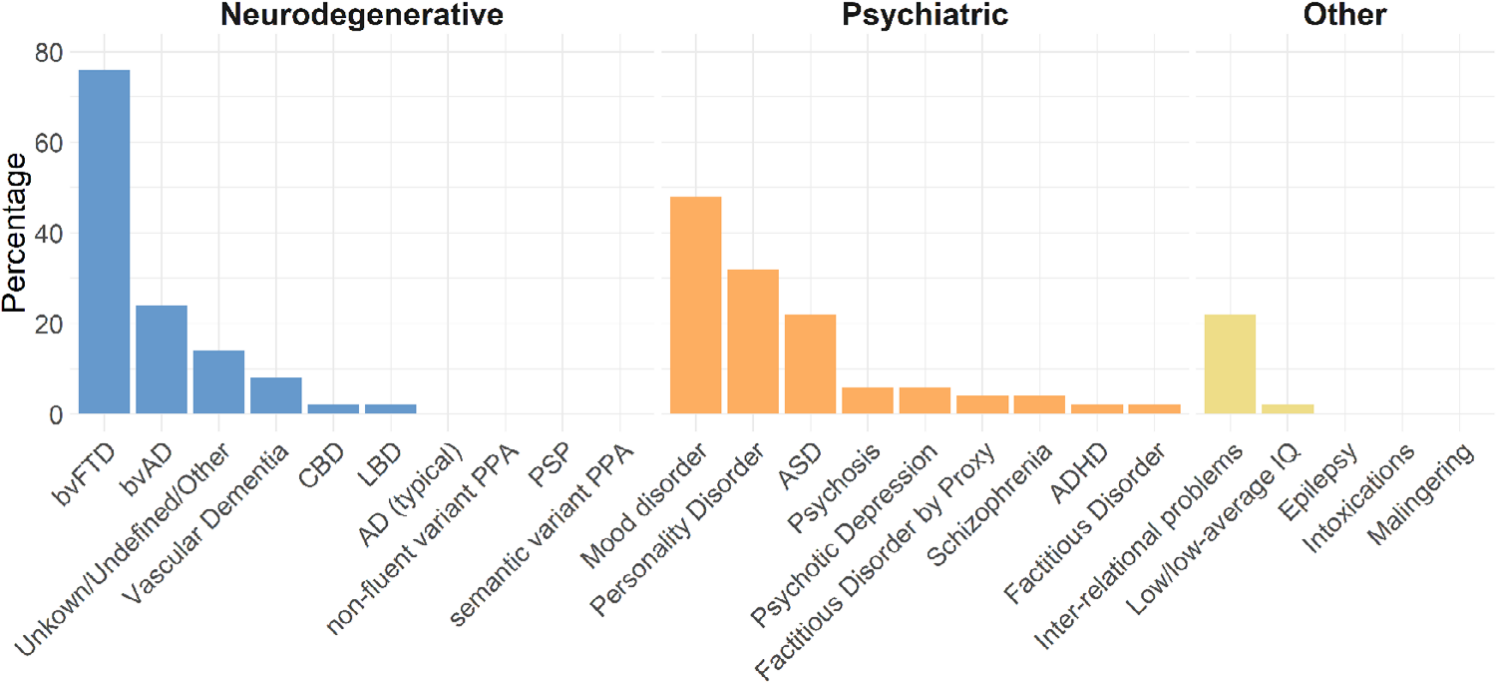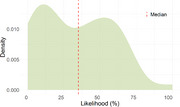# Towards uniformity in bvFTD Phenocopy Syndrome: specialists’ perspectives and proposed research criteria

**DOI:** 10.1002/alz70857_099793

**Published:** 2025-12-24

**Authors:** Paula I. Vanneste, Flora H. Duits, Willem Lucas Hartog, Dirk N. van Paassen, Marie‐Paule E. van Engelen, Dhamidhu Eratne, Simon Ducharme, Yolande A.L. Pijnenburg, Welmoed A. Krudop, Neuropsychiatric International Consortium on Frontotemporal Dementia phenocopy working group

**Affiliations:** ^1^ Alzheimer Center Amsterdam, Neurology, Vrije Universiteit Amsterdam, Amsterdam UMC location VUmc, Amsterdam, Netherlands; ^2^ Amsterdam Neuroscience, Neurodegeneration, Amsterdam, Netherlands; ^3^ Alzheimer Center Amsterdam, Department of Neurology, Amsterdam Neuroscience, Vrije Universiteit Amsterdam, Amsterdam UMC, Amsterdam, Netherlands; ^4^ Alzheimer Center Amsterdam, Amsterdam UMC location VUmc, Amsterdam, North‐Holland, Netherlands; ^5^ Amsterdam Neuroscience, Amsterdam, North‐Holland, Netherlands; ^6^ Neuropsychiatry Centre, The Royal Melbourne Hospital, Parkville, VIC, Australia; ^7^ Douglas Mental Health University Institute, Montreal, QC, Canada; ^8^ McConnell Brain Imaging Centre, Montreal Neurological Institute, McGill University, Montreal, QC, Canada; ^9^ GGZ inGeest Specialised Mental Health Care, Department of Old Age Psychiatry and Neuropsychiatry, location De Nieuwe Valerius, Amsterdam, Netherlands

## Abstract

**Background:**

The phenocopy syndrome of behavioral variant FTD (phFTD) refers to patients exhibiting clinical characteristics of bvFTD but without objective progression during follow‐up. As no diagnostic criteria currently exist, we aimed to assess clinicians’ perspectives on the diagnostic process for phFTD. By integrating these perspectives, we aim to develop research criteria for phFTD.

**Method:**

We established the Phenocopy Working Group within the Neuropsychiatric International Consortium on Frontotemporal Dementia (NIC‐FTD), a consortium of researchers with expertise in FTD and psychiatric disorders. We employed an international Delphi methodology comprising three rounds, each involving an online survey followed by a group meeting to discuss controversies. Here we present the results of the first round. We recently sent out the second‐round survey, in which we aim to reach consensus on the remaining categories, incorporating clinical presentation, diagnostic tools and follow‐up. In a third and final round we will establish consensus on the final criteria.

**Result:**

The expert panel of the first Delphi round consisted of 50 clinicians with a median of 10 (IQR: 5‐20) years of experience in phFTD. According to panelists’ estimations, Raskovsky (2011) criteria most frequently met in phFTD were apathy (90%), loss of empathy (64%) and disinhibition (60%). Additionally, memory complaints and depressive symptoms were considered often present. Genetic screening for C9orf72 was almost unanimously endorsed, as were structural MRI and [18F]‐FDG‐PET as minimally required neuroimaging (Figure 1). Most frequent differential diagnoses were bvFTD (76%), mood disorders (48%), personality disorders (32%) and bvAD (24%) (Figure 2). Two thirds of panelists presumed the likelihood of a neurodegenerative etiology for phFTD to be ≤50% (Figure 3). 85% considered clinical follow‐up of at least two years appropriate before appointing a label of phFTD.

**Conclusion:**

The first Delphi round showed expert consensus on several components of the diagnostic process for phFTD, including genetic testing for C9orf72, baseline and repeated structural MRI and FDG‐PET and follow‐up duration. Completing the full Delphi procedure will result in a set of research criteria. Establishment of these criteria will enhance accurate identification of phFTD, facilitate research and advance clinical care for this rare and poorly understood patient group.